# 7SK Acts as an Anti-tumor Factor in Tongue Squamous Cell Carcinoma

**DOI:** 10.3389/fgene.2021.642969

**Published:** 2021-04-01

**Authors:** Bowen Zhang, Sainan Min, Qi Guo, Yan Huang, Yuzhu Guo, Xiaolin Liang, Li-ling Wu, Guang-yan Yu, Xiangting Wang

**Affiliations:** ^1^Department of Geriatrics, The First Affiliated Hospital of University of Science and Technology of China, Division of Life Sciences and Medicine, University of Science and Technology of China, Hefei, China; ^2^Hefei National Laboratory for Physical Sciences at the Microscale, University of Science and Technology of China, Hefei, China; ^3^Department of Oral and Maxillofacial Surgery, Peking University School and Hospital of Stomatology, Beijing, China; ^4^Department of Physiology and Pathophysiology, Peking University School of Basic Medical Sciences, Key Laboratory of Molecular Cardiovascular Sciences, Ministry of Education, and Beijing Key Laboratory of Cardiovascular Receptors Research, Beijing, China

**Keywords:** 7SK, tongue squamous cell carcinoma, tumor suppressor, *FOXJ3*, *THRA*

## Abstract

Increasing evidence has shown the mechanistic insights about non-coding RNA 7SK in controlling the transcription. However, the biological function and mechanism of 7SK in cancer are largely unclear. Here, we show that 7SK is down-regulated in human tongue squamous carcinoma (TSCC) and acts as a TSCC suppressor through multiple cell-based assays including a migration assay and a xenograft mouse model. The expression level of 7SK was negatively correlated with the size of tumors in the 73 in-house collected TSCC patients. Through combined analysis of 7SK knockdown of RNA-Seq and available published 7SK ChIRP-seq data, we identified 27 of 7SK-regulated genes that were involved in tumor regulation and whose upstream regulatory regions were bound by 7SK. Motif analysis showed that the regulatory sequences of these genes were enriched for transcription factors *FOXJ3* and *THRA*, suggesting a potential involvement of *FOXJ3* and *THRA* in 7SK-regulated genes. Interestingly, the augmented level of *FOXJ3* in TSCC patients and previous reports on *THRA* in other cancers have suggested that these two factors may promote TSCC progression. In support of this idea, we found that 21 out of 27 aforementioned 7SK-associated genes were regulated by *FOXJ3* and *THRA*, and 12 of them were oppositely regulated by 7SK and *FOXJ3/THRA*. We also found that *FOXJ3* and *THRA* dramatically promoted migration in SCC15 cells. Collectively, we identified 7SK as an antitumor factor and suggested a potential involvement of *FOXJ3* and *THRA* in 7SK-mediated TSCC progression.

## Introduction

7SK is highly abundant and evolutionarily conserved non-coding RNA (ncRNA) in vertebrates. It is transcribed by RNA polymerase III (Pol III) and predominantly localized in the nucleus (Wassarman and Steitz, 1991). 7SK is best known for acting as a scaffold with multiple proteins to form small nuclear ribonucleoprotein (snRNP) with *LARP7, HEXIM1*, and *MEPCE*. 7SK snRNP has the ability to inhibit gene transcriptional elongation by sequestering the positive transcription elongation factor b (P-TEFb) (He et al., 2008; Abasi et al., 2016; Eichhorn et al., 2018). P-TEFb consists of CDK8 and cyclin T1 or T2 and regulates gene transcriptional elongation. Release of 7SK from snRNP complex leads to P-TEFb activation and transcriptional elongation.

Despite its well-investigated molecular mechanism in snRNP activity control, the biological function of 7SK is largely unclear. It has been shown that the expression level of 7SK was down-regulated in breast cancer, colon cancer, and Chronic Myelogenous Leukemia (CML) (Abasi et al., 2016). These reports suggested that 7SK may possess a tumor-suppressive role. Recently, an inhibitory role of LARP7 in tumor progression has been reported. In this report, knocking downLARP7 resulted in decreasing 7SK snRNP and released P-TEFb to positively regulate EMT associated genes, such as Slug, FOXC2, and ZEB2, which ultimately caused increased EMT, invasion, and metastasis in breast cancer (Ji et al., 2014). In view of the critical role of 7SK as a general regulator in gene transcription control and the suggestive dysregulation of 7SK in the previously reported cancer types, it would be of great interest to explore the biological function of 7SK in cancer and the underlying regulatory mechanism.

Tongue squamous cell carcinoma (TSCC) is a malignant tumor characterized by high incidence, mortality, and risk of metastasis to lymph node and other organs in the early stages (Annertz et al., 2012; Michikawa et al., 2012). Due to the limited understanding of the molecular mechanism, the targeted therapy is less involved for TSCC (Omura, 2014; Guo et al., 2018; Chen et al., 2020). It is urgent to explore the novel regulatory molecules to pursue potential targeted therapy development and drug discovery in TSCC. NcRNAs have been shown to participate in the regulation of TSCC progression. For example, long non-coding RNA (lncRNA) *KCNQ1OT1* is highly expressed in TSCC tissues, and knockdown of *KCNQ1OT1* repressed TSCC proliferation via miR-211-5p mediated Ezrin/Fak/Src signaling (Zhang et al., 2018). And miR-22 has been shown to be able to promote TSCC apoptosis upon *cis*-platin treatment through PI3K/Akt/NF-κB signaling (Gu et al., 2018).

Here, we showed that 7SK was down-regulated in 63% of the TSCC patients, and the expression level of 7SK was negatively correlated with the size of the tumor. We also found that knockdown of 7SK promoted cell growth and migration in cultured SCC15 cells and xenograft tumor formation in nude mice. Through RNA-seq and data mining of the reported ChIRP-seq (Flynn et al., 2016), we discovered 27 7SK-regulated genes that showed occupancy by 7SK RNA. Furthermore, we built a potential correlation between 7SK and FOXJ3/THRA through identifying FOXJ3 and THRA binding elements on the 5′ regulatory regions of these genes. Among these 27 7SK-regulated genes, 21 of them were also regulated by FOXJ3 and THRA, including 12 genes that are showed to be oppositely controlled by 7SK and FOXJ3/THRA. Interestingly, the expression level of FOXJ3 was augmented in TSCC patients and previous reports of other cancer have suggested an oncogenic role of THRA. In support of this, we also found that FOXJ3 and THRA dramatically promoted migration in SCC15 cells. All in all, we have reported a tumor-suppressive role of 7SK in TSCC and suggested a putative functional involvement of FOXJ3 and THRA in 7SK-mediated TSCC progression.

## Materials and Methods

### Cell Culture

Human tongue squamous cell carcinoma cell line SCC15 was purchased from the American Type Culture Collection (ATCC). SCC15 cells were cultured in a DMEM/F-12 medium (Sigma, United States) supplemented with 10% fetal bovine serum (FBS, Gibco, United States), 1% penicillin, and streptomycin (WISENT Inc., CA) and incubated under humidified atmosphere of 5% CO_2_ at 37°C and maintained at the confluency of approximately 80%.

### Plasmid Construction

The small hairpin RNAs (shRNAs) targeting human 7SK were subcloned into the expression vector pLKO.1 using *Age*I and *Eco*RI restriction enzymes (Thermo Fisher Scientific, United States) under the control of the U6 promoter. The resultant constructs were designated as sh7SK-1 and sh7SK-2. Sequences used for sh7SK are listed in Supplementary Table 5.

### Transfection

Cells were pre-seeded on 60 mm dishes at a density of 6 × 10^5^/dish the day before transfection. ShRNAs target 7SK or the negative control in the PLKO.1 vector and siRNAs target FOXJ3 and THRA were transfected into cells using Lipofectamine 3,000 (Invitrogen, United States) following the manufacturer’s instructions. Transfectants were collected 48 h after transfection. SiRNAs were purchased from RiboBio, and sequences used for siFOXJ3 and siTHRA are listed in Supplementary Table 5.

### RNA Extraction, Reverse Transcription (RT), and Real-Time Quantitative PCR (RT-qPCR)

Cultured cells were washed twice in ice-cold phosphate-buffered saline (PBS) and lysed with TRIzol reagent (Ambion, United States) to isolate total RNA, according to the manufacturer’s instructions. After treatment with DNase I (Thermo Fisher Scientific, United States), the first-strand cDNA was synthesized using HiScript II 1st Strand cDNA Synthesis Kit (Vazyme, CHINA). The cDNA was analyzed with real-time PCR using a SYBR Green qPCR kit (Vazyme, CHINA). All primers for each gene are listed in Supplementary Table 6. Quantitative PCR was conducted at 95°C for 5 min followed by 40 cycles of 95°C for 10 s and 60°C for 30 s in LightCycler^®^ 96 of Roche. The 2^−ΔΔCt^ method was used to quantify the relative expression using GAPDH as the endogenous control. Primers used for RT-qPCR are listed in Supplementary Table 6.

### Apoptosis Analysis

SCC15 cells were transfected with sh7SK or shRNA control, respectively. SCC15 cells were harvested by trypsinization and washed with PBS. For apoptosis analysis, 1 × 10^5^ cells were harvested and an Annexin V Staining Kit (BD Biosciences, United States) was used according to the manufacturer’s instructions 48 h after transfection. The stained cells were analyzed with Cellometer Vision Image Cytometer (Nexcelom Bioscience, United States). Data were analyzed in FCS4 Express Cytometry (*De Novo* Software, United States).

### RNA-Seq

The control and two 7SK knockdown SCC15 cell lines were collected for RNA-Seq. RNA library construction and sequencing were performed with BGISEQ-500. About ∼6 GB raw RNA-seq data were obtained for each sample. For each experiment, three biological replicates were sequenced. The differential expressed genes (DEGs) were determined by using Fold change ≥ 2 and diverge probability ≥ 0.8 as criteria. The raw data is uploaded to NCBI SRY, the accession No. is PRJNA686697.

### Patient Sample Collection

Tumor and matched non-malignant tissues of 73 patients were recruited from Peking University School and Hospital of Stomatology. All of the fresh tissue specimens were delivered under liquid nitrogen after being dissected carefully at least 1.5 cm from the tumor margins and stored at −80°C before use. The surgical samples contained at least 70% tumor or normal cells, which was confirmed by a pathologist using Hematoxylin-Eosin staining. The clinicopathological factors of patients were calculated in Supplementary Table 1. Tumor sizes and clinical stages were informed from clinical examination and radiogram and classified patients into clinical stage I–IV in terms of TNM staging system. With respect to lymph node metastasis, the status was judged according to histopathologic examination of the regional lymph nodes. The research was approved by the Ethics Committees for Human Experiments of Peking University School and Hospital of Stomatology (Approval number PKUSSIRB-2013009). All patients signed an informed consent document before tissue collection.

### Cell Migration Assay

To begin, 8 μm-pore polycarbonate membrane divided transwell chambers into upper and lower chambers. And 3.0 × 10^4^ cells resuspended with serum-free DMEM/F12 medium were added to the upper chamber and medium containing 20% FBS, which was added to the lower chamber before the chamber was incubated at 37°C in 5% CO_2_ for 24 h. After the removal of non-migrated cells on the upper side of the membrane by cotton swab, the membrane was fixed with 95% ethanol and stained with hematoxylin-eosin (ZSGB, CHINA). The migrated cells were counted using light microscopy. The experiment was performed three times independently.

### Scratch Wound Healing Assay

Cells were plated into 6-well plates and incubated at 37°C in 5% CO_2_ until reached to 100% confluence. Straight scratches were made after treating cells with 1 μg/ml mitomycin C (Roche, United States) for 2 h. After we washed it with 1 × PBS, serum-free medium was added and the cells were further cultured for 20 h. The cells from six views in each well were photographed at 0 and 20 h. The migration distance was assessed using Image-pro plus 6.0. The average distance of the six counted views was calculated and used for evaluating the wound healing effect. The wound healing experiment was performed three times independently.

### MTS Cell Proliferation Assay

Cell proliferation was assessed by CellTiter 96^®^ AQueous One Solution Cell Proliferation Assay (Promega, Madison, WI, United States), a 3-(4,5-dimethylthiazol-2-yl)-5-(3-carboxymethoxyphenyl)-2-(4-sulfophenyl)-2H-tetrazolium (MTS) assay. Squamous cell carcinoma cells were seeded in 96-well plates (2,000 cells per well). Then 20 μl MTS reagent was mixed in each well after 24 or 48 h and incubated in 37°C for 4 h before detection. The plate was measured absorbance at 490 nm.

### Nude Mice Xenograft Model

All experimental procedures were approved by the Ethics Committee of Animal Research, Peking University Health Science Center. Four female BALB/c nude mice were obtained from Peking University Health Science Center. And 6 × 10^6^ of 7SK knockdown and control cells were subcutaneously injected at the armpits. Nude mice xenograft tumor models were assessed after 3 weeks and hematoxylin-eosin (HE) staining was performed to analyze tumor pathological feature under the microscope.

### ChIRP-Seq Analysis

The 7SK ChIRP-Seq data included hg19_7SK_ChIRP-seq_HeLa.bw and hg19_7SK_ChIRP-seq_HeLa_Input.bw were obtained from Gene Expression Omnibus database under accession code GSE69141 (Flynn et al., 2016). The obtained data were uploaded to UCSC Custom Tracks and analyzed for the occupancy of 7SK on the 59 DEGs.

### Statistical Analysis

Data were presented as the mean ± SEM. Statistical analysis of the results were performed using unpaired Student’s *t-*tests with GraphPad Prism, GraphPad Software, La Jolla California, United States^1^. *P* < 0.05 was considered significant.

## Results

### Downregulation of 7SK Correlates With Tumor Size in TSCC Patients

**FIGURE 1:**
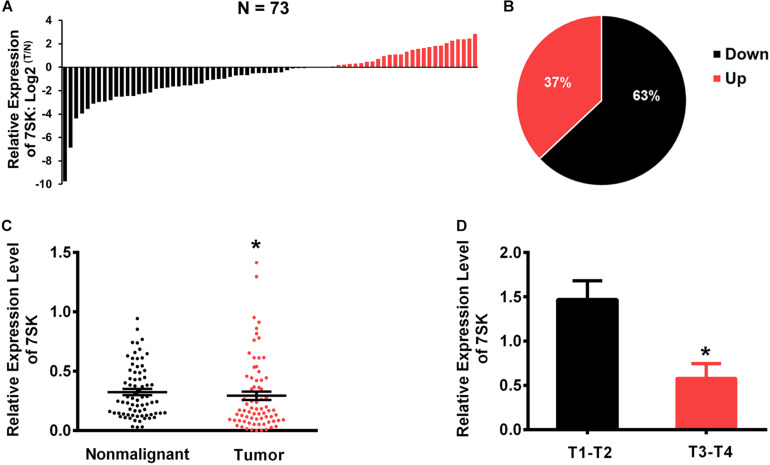
The expression levels of 7SK in TSCC patients. **(A)** The Log_2_ fold changes of 7SK expression (in tumors and non-malignant samples) in each of 73 TSCC patients. **(B)** Pie chart showing ratios of different 7SK expression in tumors and non-malignant areas of 73 patients. **(C)** Scatterplot showing the expression levels of 7SK in 73 TSCC tumors and the corresponding non-malignant samples. **(D)** The expression levels of 7SK in different T stages of the 73 patients. *P < 0.05.

To investigate whether 7SK is involved in human TSCC progression, we detected the expression level of 7SK in clinical specimens. Our results showed that the expression level of 7SK in 46 out of 73 TSCC samples (63%) were significantly decreased when compared to the matched non-malignant counterpart (Tumor/Non-malignant, T/N) (Figures 1A,B). Statistical analysis showed that the downregulation of 7SK in TSCC was significant (Figure 1C). To further establish the correlation between 7SK expression and TSCC risk, corresponding 7SK expression levels of clinicopathological features were calculated and summarized in Supplementary Table 1. Our results showed that patients with low 7SK expression had poorer T stage than those patients with higher 7SK expression (Figure 1D), indicating a negative correlation between 7SK expression and the tumor size of TSCC. Note that there is no significant difference between 7SK expression and clinicopathological factors such as age, gender, or lymph nodes metastatic (Supplementary Table 1) or prognosis of TSCC patients (Supplementary Figure 1). The association between tumors and reduced 7SK expression was further examined by using cultured tumor cell lines. Decreased expression level of 7SK was also observed in human tongue squamous carcinoma (TSCC) SCC15 cells, compared with the normal oral keratinocyte HOK cells (Supplementary Figure 2). The down-regulated 7SK in TSCC patients and SCC15 cells indicates a tumor suppressor role of 7SK. SCC15 cells were chosen as a cell model for further investigations.

### Knockdown of 7SK Leads to Increased Cell Proliferation and Migration, but Decreased Apoptosis in SCC15 Cell–

**FIGURE 2:**
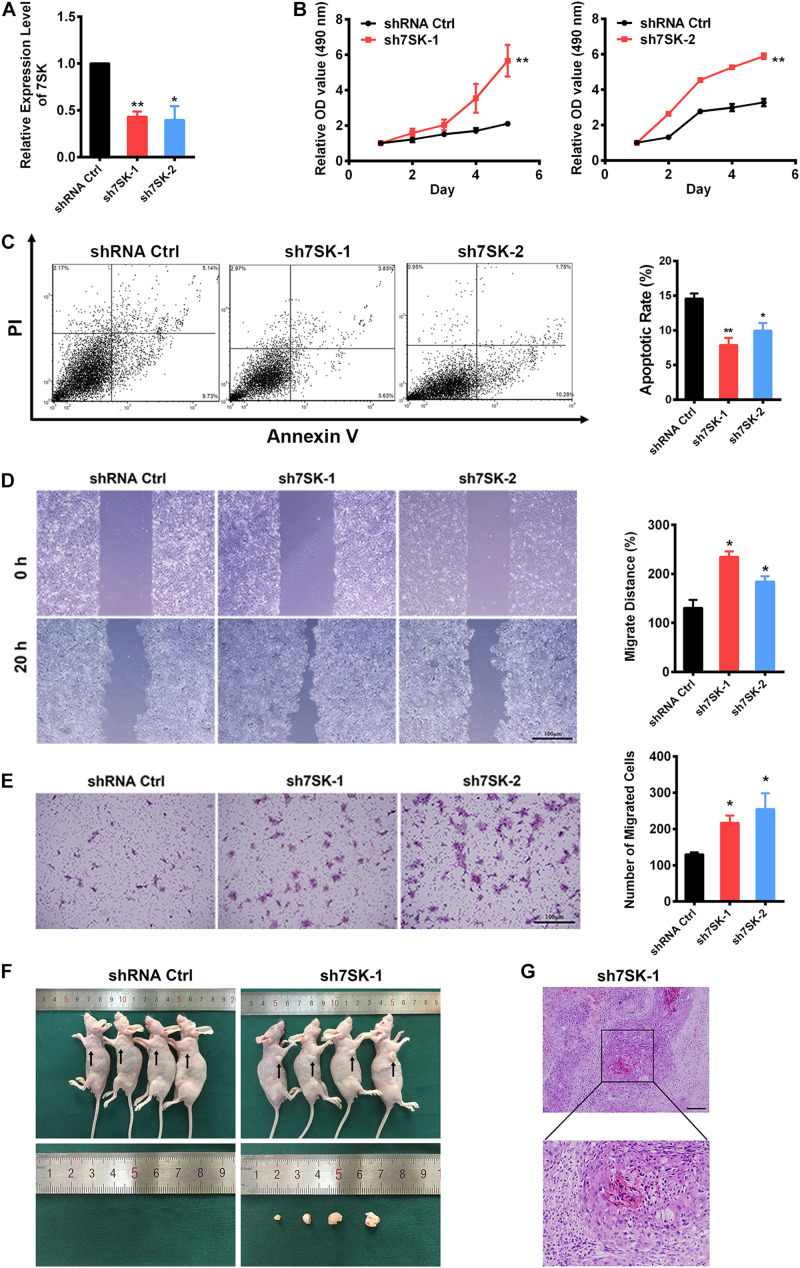
7SK inhibits tumor progression *in vitro* and *in vivo*. **(A)** Efficiency of 7SK knockdown in SCC15 cells, *n* = 3. **(B–E)** Apoptosis assay detected by Annexin V/PI staining **(B)**, MTS assay **(C)**, wound healing assay **(D)**, and transwell assay **(E)** in 7SK knockdown SCC15 cells, *n* = 3. For **(C–E)**, the representative images and corresponding statistical analysis are shown. The scale bar is 100 μm. **(F)** Images of nude mice injected with control or sh7SK-1 SCC15 cells, as well as the lumps from the injection of sh7SK-1 SCC15 cells. **(G)** HE staining of lumps from sh7SK-1 SCC15 cells injection, *n* = 4. The scale bar is 50 μm. Data represent the mean ± SEM. *P < 0.05. **P < 0.01.

To pursue the biological role of 7SK in SCC15 cell, we generated two 7SK shRNAs. Both 7SK shRNAs showed more than 50% knockdown efficiency (Figure 2A). To test the effects of 7SK on SCC15 proliferation, cell death or migration, we performed MTS assay, apoptotic assay detected by Annexin V/PI staining, wound healing assay, and transwell assay (Figures 2B–E and Supplementary Figure 3). Apoptotic, wound healing, and transwell assays were performed in the presence of transiently transfected 7SK shRNAs. For MTS assay, we used the most efficient single stable clones among the tested sh7SK-1 and sh7SK-2 transfectants (Supplementary Figure 4). Compared with the control shRNA transfected SCC15 cells, 7SK shRNAs significantly enhanced SCC15 cell proliferation detected by MTS assay (Figure 2B) but markedly reduced apoptotic rate (Figure 2C). In addition, 7SK shRNAs markedly fastened wound closure rate in wound healing assay (Figure 2D), and possessed higher migration in transwell assay (Figure 2E). Consistently with the enhanced effect of 7SK knockdown in transwell assay, our data showed that overexpression of 7SK significantly decreased the cell migration rate in SCC15 cells (Supplementary Figures 3A,B).

### 7SK Deficiency of SCC15 Cells Strongly Induces Tumor Formation in Nude Mice

To determine the *in vivo* role of 7SK, we conducted nude mice xenograft assay by using the same sh7SK-1 stable clone for the aforementioned MTS assay. The control shRNA or sh7SK-1 cells were injected subcutaneously into the armpits of four nude mice and were examined 3 weeks later. Consistently with our prediction, in the right armpits transplanted with sh7SK cells we observed obvious lumps formation while the left armpits transplanted with control shRNA did not form any lump during the observed time window (Figure 2F). All transplanted nude mice developed progressive cachexia, and the skin area of the sh7SK lumps festered. HE staining of the sh7SK lump tissues showed abnormal mitoses, high atypia, and nests composed of polygonal cells, which are typical characteristics of squamous cell carcinoma (Figure 2G). All in all, our results suggest that knockdown of 7SK promoted cell growth and migration in cultured SCC15 cells, and xenograft tumor formation in nude mice.

### RNA-Seq Reveals 7SK Regulated Genes That Are Enriched in Cell Proliferation, Migration, and Cell Death in SCC15 Cells

**FIGURE 3:**
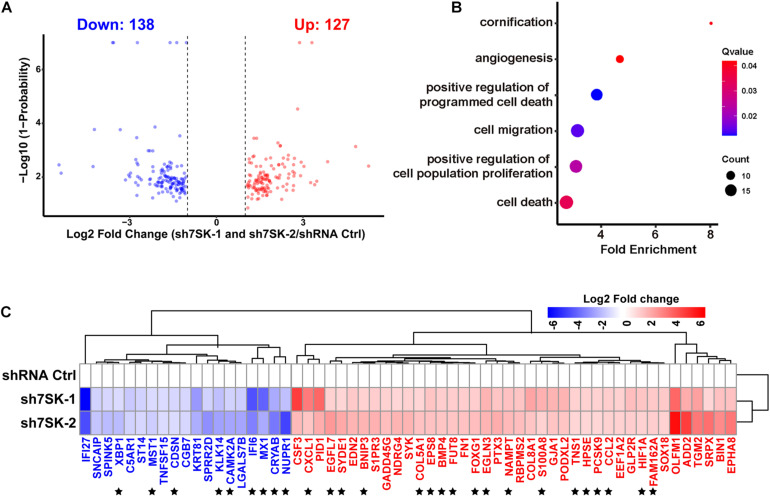
7SK regulates genes that are involved in tumor progression. **(A)** Volcano plot of commonly regulated genes identified from sh7SK-1 and sh7SK-2 RNA-seq compared with shRNA Ctrl. **(B)** Biological processes related to tumor progression and significantly enriched by Gene Ontology (GO) analysis using 7SK regulated genes from **(A)**. **(C)** The Log_2_ fold changes of the genes that are identified from **(B)** are illustrated on a heat map. ★, 27 genes that are further identified as 7SK directly associated genes shown in Figure 4. Blue: down-regulated genes in the presence of 7SK shRNAs; red: up-regulated genes in the presence of 7SK shRNAs, *n* = 3.

To systematically identify downstream genes regulated by 7SK, we performed RNA sequencing (RNA-Seq) for 7SK knockdown SCC15 cells with two shRNAs. Analyses on the size of clean reads, mapping rates, and quality control have confirmed the suitable quality for the obtained RNA-Seq data (Supplementary Tables 2–4). Correlation analysis shown in Supplementary Figure 5 indicated that all triplicates in each treatment are comparable and reproducible. A total of 127 up-regulated genes and 138 down-regulated genes were identified (Figure 3A and Supplementary Table 7). In concert with the enhanced tumor progression in 7SK knockdown SCC15 cells, 40 of 127 up-regulated genes were found to be enriched in gene ontology (GO) terms that were involved in proliferation, migration, and angiogenesis; while 19 of the 138 down-regulated genes were enriched for cell death and cornification by GO analysis (Figure 3B). A heatmap of these 59 genes (40 up-regulated and 19 down-regulated) is shown in Figure 3C. Among these, 27 genes (star-marked in Figure 3C) have been further identified to have occupancy with 7SK RNA (detailed explanation is provided under the headline “ChIRP-Seq Data Mining Reveals PAX5, THRA, FOXJ3, and Two Novel Motifs on 7SK-Associated Genes”), and validated by RT-qPCR (Supplementary Figure 6).

### ChIRP-Seq Data Mining Reveals PAX5, THRA, FOXJ3, and Two Novel Motifs on 7SK-Associated Genes

**FIGURE 4:**
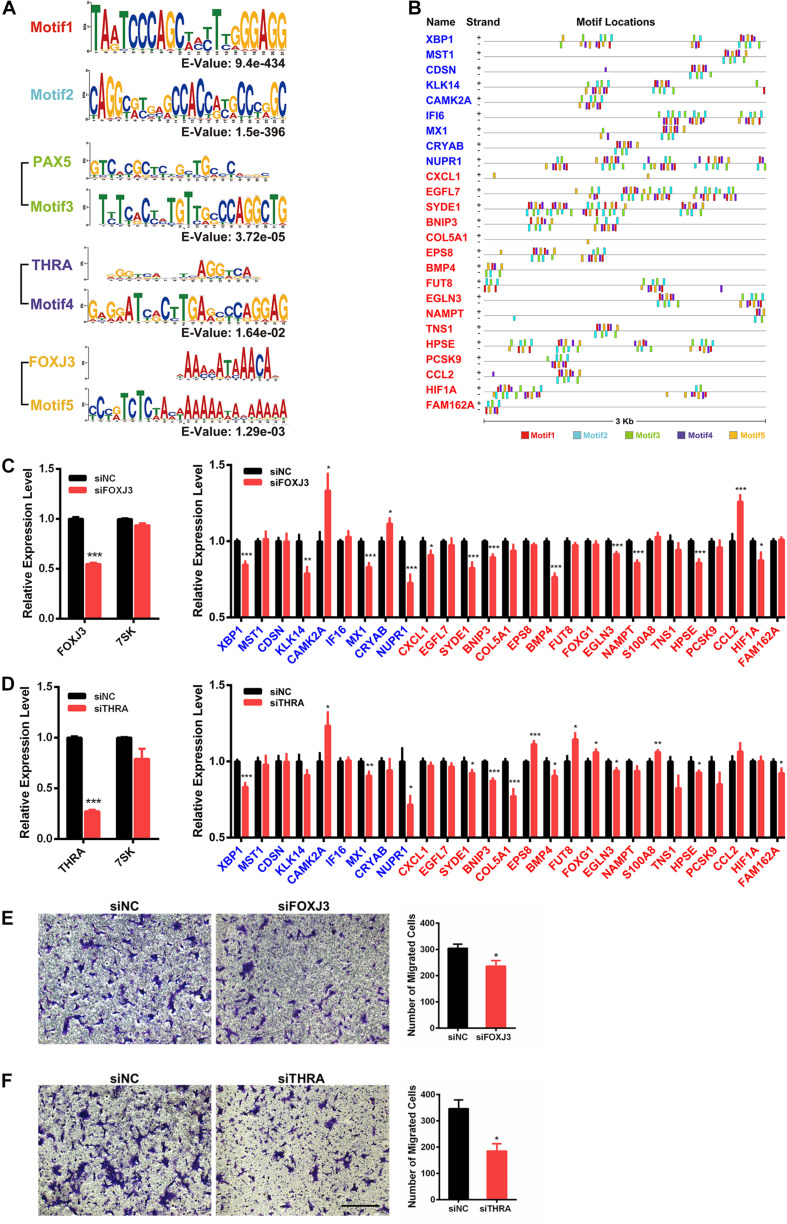
Identification of *FOXJ3* and *THRA* as two transcription factors that oppositely regulate 7SK associated genes and SCC15 migration. **(A)** The top five motifs (including *FOXJ3* and *THRA*) identified on the upstream regulatory regions of the 27 7SK associated genes. **(B)** Motif distributions on the upstream regulatory regions of the 27 7SK-associated genes. **(C,D)** RT-qPCR of *FOXJ3*, *THRA*, 7SK, and the 27 7SK-associated genes in the presence of siFOXJ3 **(C)** and siTHRA **(D)** treated SCC15 cells. **(E,F)** The representative images (left) and the according statistical analysis (right) of transwell assay after *FOXJ3* **(E)** and *THRA* **(F)** knockdown. The scale bar is 100 μm, *n* = 3. *P < 0.05.

To investigate if 7SK has physical interactions with the 5′ regulatory regions of the above identified 59 7SK-regulated genes to regulate the expression, we took advantage of the 7SK ChIRP-Seq data published by Chang lab (Flynn et al., 2016). Our data mining results indicated that 27 of the 59 genes showed occupancy of 7SK (Supplementary Figure 7, and star-marked in Figure 3C). To further investigate the potential regulatory mechanism of these 27 7SK-associated genes, we conducted a bioinformatic analysis to explore the common motifs within the 5′ regulatory regions of these 27 genes up to 3 Kb upstream from their transcription start sites and obtained 5 motifs. Then, we performed motif analysis on the five motifs, and we found that motifs 1 and 2 do not match any known binding elements to the reported transcription factors, while motifs 3, 4, and 5 possess similar DNA binding motifs to *PAX5*, *THRA*, and *FOXJ3*, respectively (Figure 4A). The distribution maps of the 5 motifs on the upstream regulatory regions of the 27 7SK-associated genes are shown in Figure 4B.

### 7SK-Associated Genes Are Regulated by FOXJ3 and THRA

Next, we attempted to identify the factors that may participate in the regulation of the 7SK-associated genes in TSCC. Our RT-qPCR results showed that PAX5 is barely detectable in SCC15 cells; in contrast, both FOXJ3 and THRA are relatively abundantly expressed in SCC15 cells (Supplementary Figure 8A). Therefore, we focused on FOXJ3 and THRA for further investigation. We found that FOXJ3 was significantly upregulated in TSCC patients, and THRA was constantly expressed in normal and TSCC tissues from TCGA database (Supplementary Figure 8B). Our RT-qPCR results showed that 21 out of these 27 genes exhibited significant changes in the presence of either knockdown of *FOXJ3 (XBP1, KLK14, CAMK2A, MX1, CRYAB, NUPR1, CXCL1, SYDE1, BNIP3, BMP4, EGLN3, NAMPT, HPSE, CCL2*, and *HIF1A)* or *THRA (XBP1, CAMK2A, MX1, NUPR1, SYDE1, BNIP3, COL5A1, EPS8, BMP4, FUT8, FOXG1, EGLN3, S100A8, HPSE*, and *FAM162A*). Among these 21 *FOXJ3-* and *THRA-*regulated genes, 9 of them (*XBP1, CAMK2A, MX1, NUPR1, SYDE1, BNIP3, BMP4, EGLN3*, and *HPSE*) are common target genes shared by FOXJ3 and THRA (Figures 4C,D). Interestingly, among these 21 genes, 12 genes (57%) are oppositely regulated by 7SK and *FOXJ3*/*THRA*. Particularly, four genes that positively regulate tumor migration are identified to be negatively regulated by 7SK but positively regulated by *FOXJ3* and *THRA*. These four genes are *CXCL1*, *SYDE1*, *COL5A1*, and *HIF1A* (Lo et al., 2017; Byun et al., 2019; Feng et al., 2019; Yang et al., 2019). Consistently with these findings, knockdown of either *FOXJ3* or *THRA* significantly decreased the cell migration rate in SCC15 cells (Figures 4E,F), which is opposite to 7SK.

Altogether, our results suggest a putative functional involvement of the transcriptional factors *FOXJ3* and *THRA* in the 7SK-associated gene expression control and tumor migration in TSCC.

## Discussion

Currently, 7SK is considered a paradigm in RNA-regulated transcription (Diribarne and Bensaude, 2009). However, the biological function of 7SK is largely unclear. Here, we identified 7SK acting as a novel tumor suppressor in TSCC, and suggested a putative functional involvement of FOXJ3 and THRA in 7SK-mediated TSCC progression.

In TSCC, we found that 7SK was down-regulated in 63% of the 73 TSCC patients, and the down-regulated 7SK is correlated with the size of tumor. In functional assays, knockdown of 7SK in cell and xenograft experiments showed a tumor suppressor role of 7SK in TSCC. Numerous reports have linked non-coding RNAs with cancer (Wapinski and Chang, 2011; Wang et al., 2019; Huang et al., 2020). For example, *HOTAIR* is found to be associated with tumor metastasis in breast cancer through binding with polycomb repressive complex 2 (PRC2) and the lysine-specific demethylase1 (LSD1) to regulate gene expression (Burd et al., 2010; Tsai et al., 2010). Our previous work showed that ncRNA*_*CCND*__1_*s bind to FUS/TLS, thus causing an allosteric effect and inhibiting the histone acetyltransferase activity of CBP/P300, leading to gene transcription suppression of *CCND1* in Hela cells (Wang et al., 2008). Interestingly, ncRNA*_*CCND*__1_*s can crosstalk with another lncRNA, namely, LINC00473, to fine-tune the expression level of *CCND1* in breast cancer cells (Shi and Wang, 2019).

*FOXJ3* (Forkhead box protein J3), *THRA* (thyroid hormone receptor alpha), and *PAX5* (paired box protein Pax-5) were identified through motif comparison analysis with 7SK binding genes. Due to the barely detectable expression level of *PAX5* in SCC15 cells, we chose not to focus on *PAX5* in the current study. However, we do not exclude the potential regulatory role of *PAX5* in 7SK-associated genes in other types of cancer. *FOXJ3* is a transcription factor that is down-regulated in colorectal cancer and lung cancer (Ma et al., 2016; Chen et al., 2018). In TSCC, the expression level of *FOXJ3* is up-regulated. *THRA* is a nuclear receptor that binds to T3 (Thyroxine 3) (Markossian et al., 2018). Interestingly, although the expression level of *THRA* has not been found to be changed in TSCC patients, our analysis, using TCGA database derived head and neck squamous cancer (HNSC) patients, found certain mutations located in the ligand-binding domain and zinc finger domain of *THRA*. These findings suggest that possible dysregulations of *THRA* in cancer may involve the changes of its DNA binding or ligand-receptor interaction abilities. *THRA* has been shown to be an adverse prognostic signature for breast cancer (Wu et al., 2020). The up-regulation of *FOXJ3* in multiple types of cancers and the link between *THRA* and the breast cancer prognostic prediction suggest oncogenic functions of these two transcription factors, which is opposite to that of 7SK. Supporting this, our results from transwell assay have demonstrated opposite roles of *FOXJ3* and *THRA* in TSCC to 7SK. In addition, we have also identified a set of genes that are regulated in the opposite way by 7SK and *FOXJ3*/*THRA*, including four positive regulators in tumor migration (*CXCL1*, *SYDE1*, *COL5A1*, and *HIF1A*). It is likely that 7SK and *FOXJ3*/*THRA* possess competitive bindings on their commonly regulated genes to control the targeted genes’ activation and repression at the transcription level. It would be of great interest to demonstrate whether a directly functional link between *FOXJ3*/*THRA* and 7SK is involved in 7SK-mediated genes and 7SK-mediated tumor progression events in the near future.

In summary, the identification of 7SK in controlling TSCC progression expands our understanding of ncRNAs in cancer biology. Our work provides a potential area of developing novel diagnosis and therapeutic markers for TSCC.

## Data Availability Statement

The datasets generated for this study can be found in the NCBI Bioproject: https://www.ncbi.nlm.nih.gov/bioproject/686697, accession PRJNA686697.

## Ethics Statement

The studies involving human participants were reviewed and approved by the Ethics Committees for Human Experiments of Peking University School and Hospital of Stomatology (Approval number PKUSSIRB-2013009). The patients/participants provided their written informed consent to participate in this study. The animal study was reviewed and approved by Ethics Committee of Animal Research, Peking University Health Science Center.

## Author Contributions

XW initiated the project and supervised the whole project. SM, LW, and GY contributed to the xenograft experiments and the experiments using TSCC patient samples. BZ, YG, and XL performed the cell-based assays. QG, YH, and BZ performed the RNA-seq and data analysis. BZ conducted all the other experiments and data analysis. BZ and XW wrote the manuscript. All authors read and accepted the final version.

## Conflict of Interest

The authors declare that the research was conducted in the absence of any commercial or financial relationships that could be construed as a potential conflict of interest. The reviewer XJ declared a shared affiliation, with no collaboration, with several of the authors SM, LW, GY to the handling editor at the time of the review.

## Footnotes

1 www.graphpad.com
