# Supplementary material for: 7SK Acts as an Anti-tumor Factor in Tongue Squamous Cell Carcinoma
Source: Front Genet. 2021 Apr 1;12:642969. doi: 10.3389/fgene.2021.642969 (PMC8047107; doi:10.3389/fgene.2021.642969)
Supplement: Supplementary file 1 [file Table_1.DOCX]

Supplementary Tables

**Supplementary Table 1. Clinical characteristics and the 7SK expression levels in the 73 TSCC patients.**

| **Characteristics** | **Total n = 73 (%)** | **Fold change of 7SK** | **P-value** |
| --- | --- | --- | --- |
| *Sex* |  |  | 0.7643 |
| Male | 43 (58) | 1.344 ± 0.2385 |  |
| Female | 30 (41) | 1.234 ± 0.2725 |  |
|  |  |  |  |
| *Age* |  |  | 0.1137 |
| < 60 | 49 (67) | 1.497 ± 0.2393 |  |
| ≥ 60 | 24 (33) | 0.895 ± 0.2210 |  |
|  |  |  |  |
| *Pathologic T stage* |  |  | 0.0497 |
| T1-T2 | 59 (81) | 1.469 ± 0.2120 |  |
| T3-T4 | 14 (19) | 0.582 ± 0.1642 |  |
|  |  |  |  |
| *Stage at Diagnosis* |  |  | 0.7760 |
| I-II | 34 (47) | 1.244 ± 0.2396 |  |
| III-IV | 39 (53) | 1.347 ± 0.2636 |  |
|  |  |  |  |
| *Lymph nodes metastatic* |  |  | 0.6544 |
| Yes | 38 (52) | 1.377 ± 0.2689 |  |
| No | 35 (48) | 1.215 ± 0.2344 |  |

Fold change of 7SK: Ratio of 7SK expression in tumor and matched nonmalignant tissues of 73 patients (mean ± SEM).

**Supplementary Table 2. Summary of RNA-seq raw and clean data.**

| **Sample** | **Raw Data Size (bp)** | **Raw Reads Number** | **Clean Data Size (bp)** | **Clean Reads Number** | **Clean Data Rate (%)** |
| --- | --- | --- | --- | --- | --- |
| shRNA Ctrl-1 | 8.98E+08 | 17960337 | 8.9E+08 | 17809559 | 99.16 |
| shRNA Ctrl-2 | 9.87E+08 | 19741659 | 9.81E+08 | 19617140 | 99.36 |
| shRNA Ctrl-3 | 1.05E+09 | 20924137 | 1.03E+09 | 20605223 | 98.47 |
| sh7SK1-1 | 1.19E+09 | 23710662 | 1.17E+09 | 23460042 | 98.94 |
| sh7SK1-2 | 1.04E+09 | 20731784 | 1.02E+09 | 20497467 | 98.86 |
| sh7SK1-3 | 1.21E+09 | 24140442 | 1.15E+09 | 22940813 | 95.03 |
| sh7SK2-1 | 1.21E+09 | 24140847 | 1.19E+09 | 23724412 | 98.27 |
| sh7SK2-2 | 1.21E+09 | 24140960 | 1.18E+09 | 23599235 | 97.75 |
| sh7SK2-3 | 1.21E+09 | 24141150 | 1.18E+09 | 23660407 | 98 |

**Clean Data Rate (%) = Clean Reads Number/Raw Reads Number**

**Supplementary Table 3. Alignment statistics of reads align to reference genome.**

| **Sample** | **Total Reads** | **Total Mapped Reads (%)** | **Unique Match (%)** | **Multi-position Match (%)** |
| --- | --- | --- | --- | --- |
| shRNA Ctrl-1 | 17809559 | 88.1 | 58.07 | 30.03 |
| shRNA Ctrl-2 | 19617140 | 87.69 | 57.02 | 30.67 |
| shRNA Ctrl-3 | 20605223 | 88.45 | 57.34 | 31.11 |
| sh7SK1-1 | 23460042 | 86.84 | 54.91 | 31.93 |
| sh7SK1-2 | 20497467 | 89.7 | 59.28 | 30.42 |
| sh7SK1-3 | 22940813 | 86.05 | 55.87 | 30.18 |
| sh7SK2-1 | 23724412 | 86.76 | 57.35 | 29.41 |
| sh7SK2-2 | 23599235 | 87.36 | 57.94 | 29.42 |
| sh7SK2-3 | 23660407 | 87.49 | 58.7 | 28.79 |

**Total Mapped Reads (%) = Unique Match (%) + Multi-position Match (%).**

**Supplementary Table 4. Summary of RNA-seq quality control items.**

| **Sample** | **Clean Read1 Q20 (%) >= 90** | **Gene Unique Mapping Ratio (%) >= 80** | **Genome Mapping Ratio (%) >= 50** |
| --- | --- | --- | --- |
| shRNA Ctrl-1 | 93.0 (Y) | 92.06 (Y) | 88.1 (Y) |
| shRNA Ctrl-2 | 92.5 (Y) | 92.39 (Y) | 87.69 (Y) |
| shRNA Ctrl-3 | 93.0 (Y) | 92.80 (Y) | 88.45 (Y) |
| sh7SK1-1 | 92.2 (Y) | 92.67 (Y) | 86.84 (Y) |
| sh7SK1-2 | 94.0 (Y) | 92.81 (Y) | 89.7 (Y) |
| sh7SK1-3 | 92.0 (Y) | 93.54 (Y) | 86.05 (Y) |
| sh7SK2-1 | 92.4 (Y) | 92.81 (Y) | 86.76 (Y) |
| sh7SK2-2 | 92.8 (Y) | 92.81 (Y) | 87.36 (Y) |
| sh7SK2-3 | 92.8 (Y) | 92.60 (Y) | 87.49 (Y) |

**“Y” means sample passed this quality control item and “N” means failed.**

**Supplementary Table 5. Sequences of shRNAs and siRNAs.**

| **Name Sequences** | |
| --- | --- |
| sh7SK1  sh7SK2  siFOXJ3  siTHRA | GACTCCAGACACATCCAAA  GCTCTCAAGGTCCATTTGTAG  GAGAGCAGCCTAACGTCTA  CTACCGCTGTATCACTTGT |

**Supplementary Table 6. Primers used for RT-qPCR.**

| **Name Sequences** | |
| --- | --- |
| GAPDH  7SK  XBP1  MST1  CDSN  KLK14  CAMK2A  IFI6  MX1  CRYAB  NUPR1  CXCL1  EGFL7  SYDE1  BNIP3  COL5A1  EPS8  BMP4  FUT8  FOXG1  EGLN3  NAMPT  S100A8  TNS1  HPSE  PCSK9  CCL2  HIF1A  FAM162A | Forward: ACATCATCCCTGCCTCTACTG  Reverse: CCTGCTTCACCACCTTCTTG  Forward: GGGTTGATTCGGCTGATCT  Reverse: CTCCTCTATCGGGGATGGTC  Forward: AAAGCTGTTGTCGGAGAGAAG  Reverse: CACAATCGTTATGCCCTCGG  Forward: AAGCCGCAGTTCACGTTTAC  Reverse: GGGTCCATCGTGTAGCACC  Forward: TCTCAGACCCCTGTAAGGACC  Reverse: CGTTCCTGGCTTAAAAGATCCTG  Forward: GGCGGGAAGGACTCTTGTC  Reverse: GCTTCTGTACTTGCACAGGTT  Forward: ACCACTACCTGATCTTCGACC  Reverse: CCGCCTCACTGTAATACTCCC  Forward: GGTCTGCGATCCTGAATGGG  Reverse: TCACTATCGAGATACTTGTGGGT  Forward: GTTTCCGAAGTGGACATCGCA  Reverse: CTGCACAGGTTGTTCTCAGC  Forward: CCTGAGTCCCTTCTACCTTCG  Reverse: CACATCTCCCAACACCTTAACTT  Forward: CATGCCTATGCCCACTTCAC  Reverse: CTTCTCTCTTGGTGCGACCTT  Forward: CTGGCGGATCCAAGCAAATG  Reverse: GCCCCTTTGTTCTAAGCCAG  Forward: TGAATGCAGTGCTAGGAGGG  Reverse: GCACACAGAGTGTACCGTCT  Forward: CAGAAGTGCGTTGGGCAGAT  Reverse: ACCACCTTATACAGGGGTTGG  Forward: CAGGGCTCCTGGGTAGAACT  Reverse: CTACTCCGTCCAGACTCATGC  Forward: TACAACGAGCAGGGTATCCAG  Reverse: ACTTGCCATCTGACAGGTTGA  Forward: TGAATGGCTACGGATCATCACC  Reverse: CACTGTCCCGTGCATAATTCT  Forward: AAAGTCGCCGAGATTCAGGG  Reverse: GACGGCACTCTTGCTAGGC  Forward: GAATCTCAGAATTGGCGCTATGC  Reverse: GAAGCTCGACCACTTGAACAT  Forward: CCGCACCCGTCAATGACTT  Reverse: CCGTCGTAAAACTTGGCAAAG  Forward: CTGGGCAAATACTACGTCAAGG  Reverse: GACCATCACCGTTGGGGTT  Forward: AATGTTCTCTTCACGGTGGAAAA  Reverse: ACTGTGATTGGATACCAGGACT  Forward: ATGCCGTCTACAGGGATGAC  Reverse: ACTGAGGACACTCGGTCTCTA  Forward: AGCGGAGACCTGACATCAC  Reverse: CGGTTTCCCTTGTTGTGTAGAAC  Forward: TCATCAATGGGTCGCAGTTAGG  Reverse: TTAGCCGTCTTTCTTCGAGGC  Forward: AGACCCACCTCTCGCAGTC  Reverse: GGAGTCCTCCTCGATGTAGTC  Forward: GAAAGTCTCTGCCGCCCTT  Reverse: GGTGACTGGGGCATTGATTG  Forward: CTGTATGGTCAGCTCAGCCC  Reverse: GGCTGTCAGACCCGAAAAGA  Forward: GTCAGGTCGCTTCAAAAAGGA  Reverse: AGATAGCTGATCTTCACTCGCAT |
