# Supplementary material for: 7SK Acts as an Anti-tumor Factor in Tongue Squamous Cell Carcinoma
Source: Front Genet. 2021 Apr 1;12:642969. doi: 10.3389/fgene.2021.642969 (PMC8047107; doi:10.3389/fgene.2021.642969)
Supplement: Supplementary file 3 [file Data_Sheet_1.pdf]

Supplementary Figures

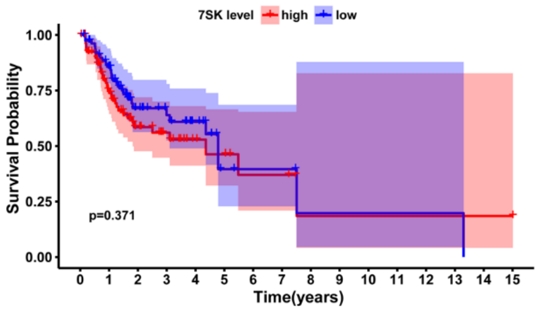

Supplementary Figure 1. Survival analysis of 7SK in TSCC patients from TCGA database.

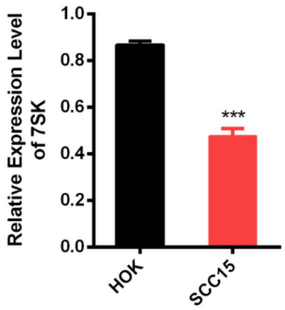

Supplementary Figure 2. Expression level of 7SK in human tongue squamous carcinoma SCC15 cells and normal oral keratinocyte HOK cells. $n = 3$. Data represent the mean ± SEM. ***$P < 0.001$.

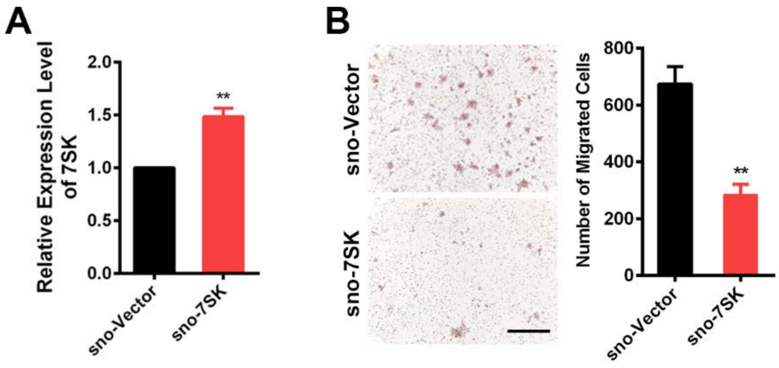

Supplementary Figure 3. (A) Expression of 7SK after 7SK overexpression. **(B)** The representative images (left) and the according statistical analysis (right) of transwell assay in 7SK overexpressed SCC15 cell. $n = 3$. The scale bar is 100 µm. Data represent the mean ± SEM. $^{**}P < 0.01$.

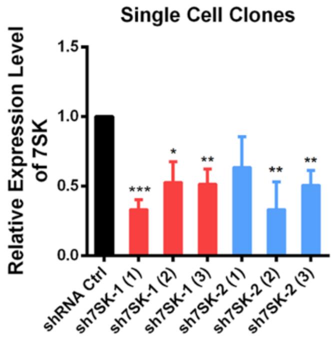

Supplementary Figure 4. Expression level of 7SK in different 7SK shRNA single cell clones. $n = 3$. Data represent the mean ± SEM. $^{*}P < 0.05$, $^{**}P < 0.01$, $^{***}P < 0.001$.

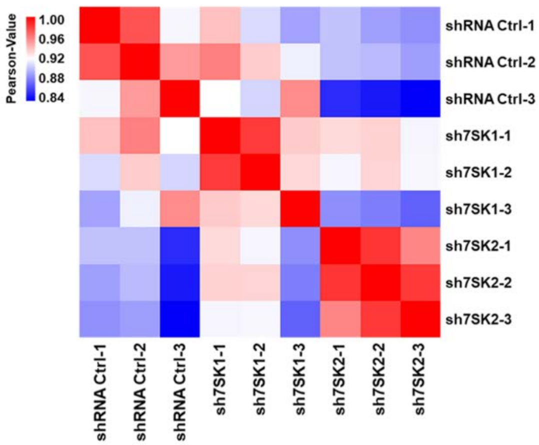

Supplementary Figure 5. Pearson correlation analysis shows the reproducible between samples of 7SK knockdown RNA-seq in SCC15.

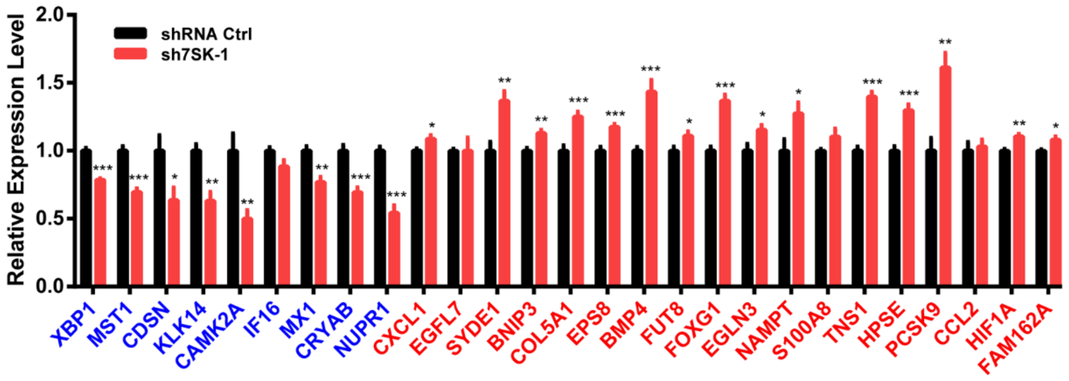

Supplementary Figure 6. Validation of the 27 star-marked (★) 7SK-associated genes by RT-qPCR. Blue, downregulated genes in the presence of 7SK shRNAs; red, upregulated genes in the presence of 7SK shRNAs. *n* = 3. Data represent the mean ± SEM. *$P < 0.05$, **$P < 0.01$, ***$P < 0.001$.

IF127:

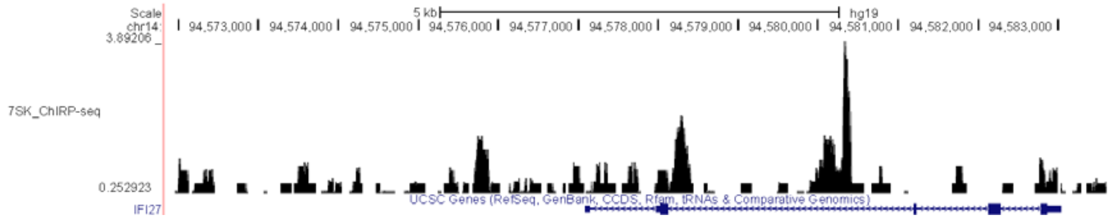

SNCAIP:

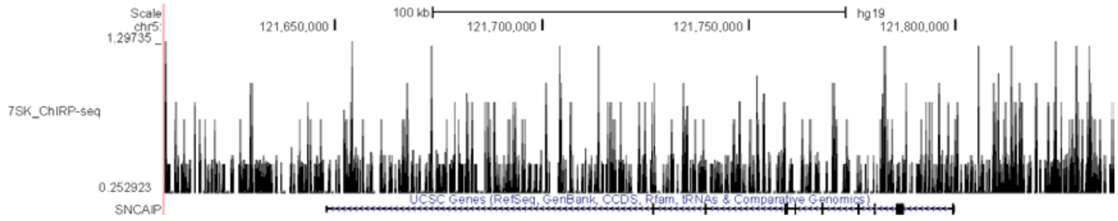

SPINK5:

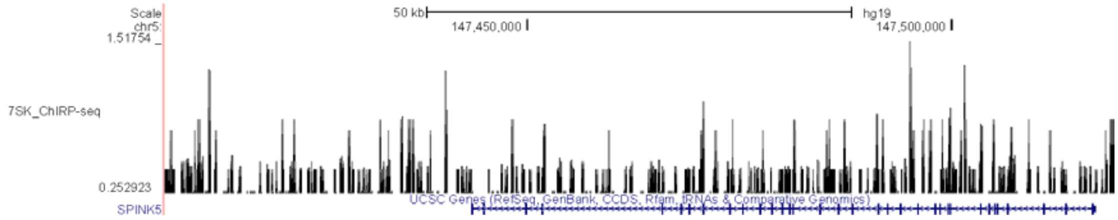

XBP1:

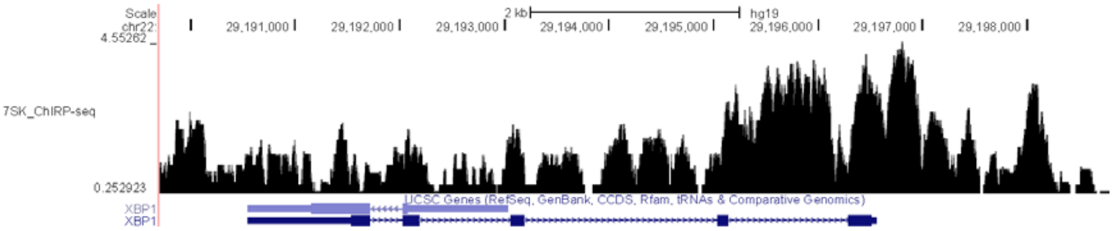

C5AR1:

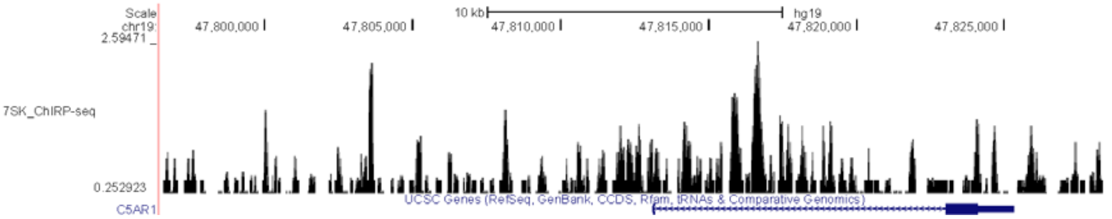

ST14:

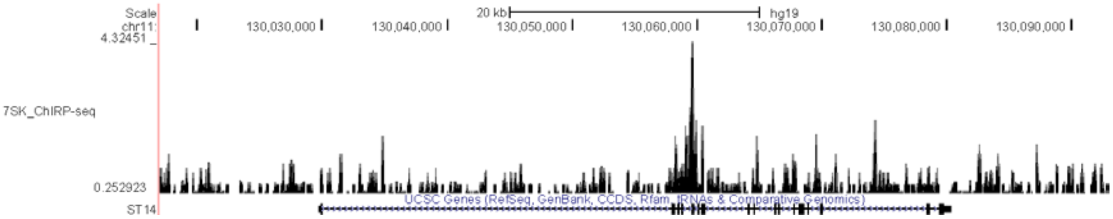

MST1: (★)

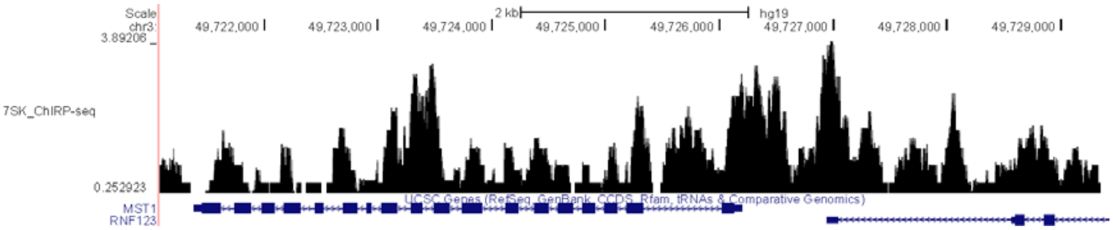

TNFSF15:

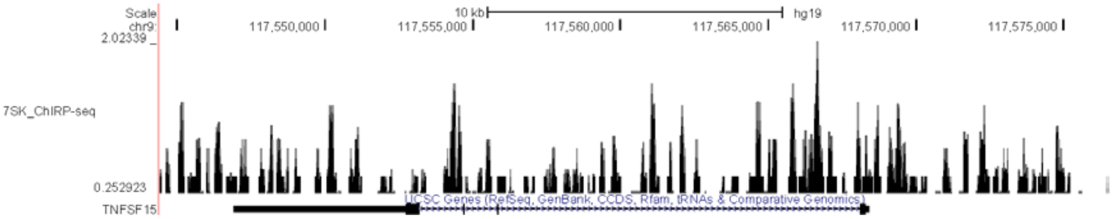

CDSN: (★)

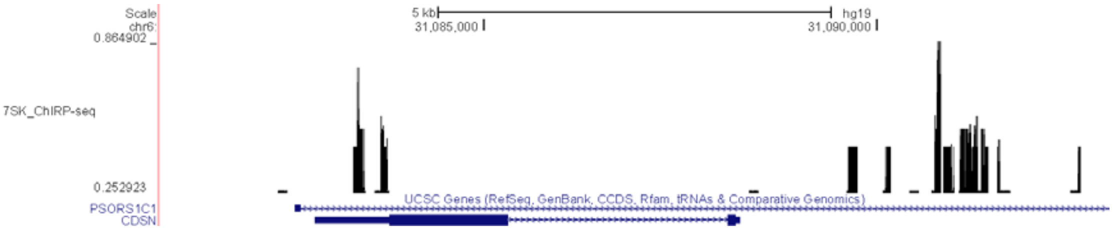

CGB7:

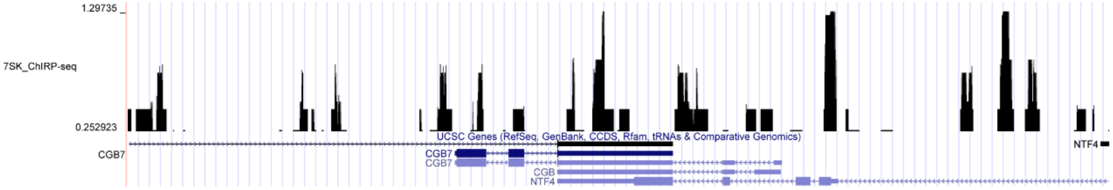

KRT81:

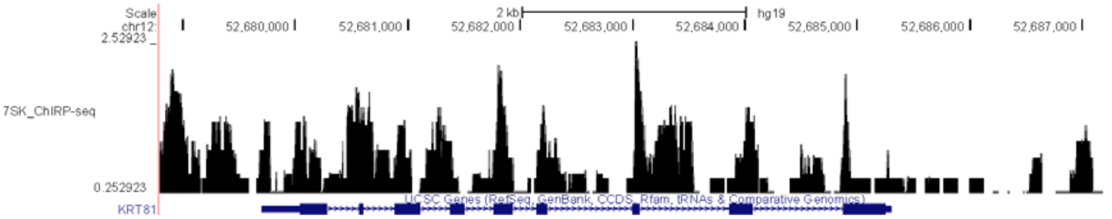

SPRR2D:

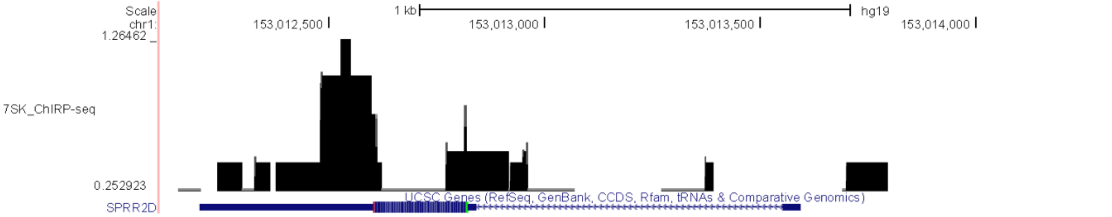

KLK14: (★)

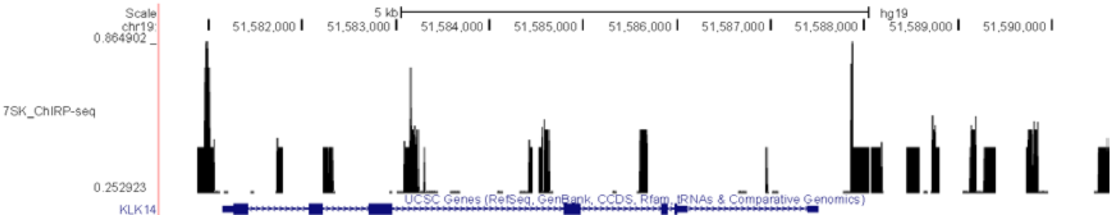

CAMK2A: (★)

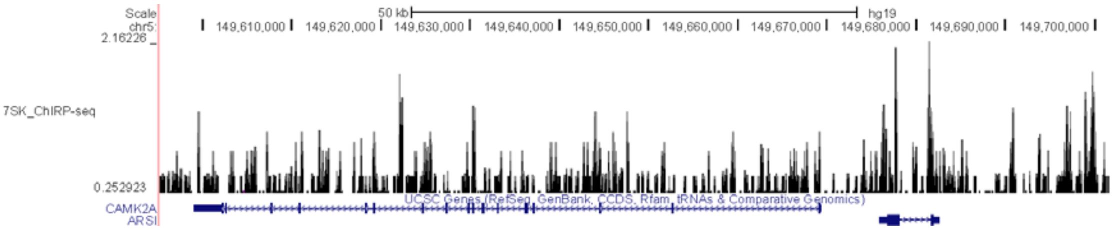

LGALS7B:

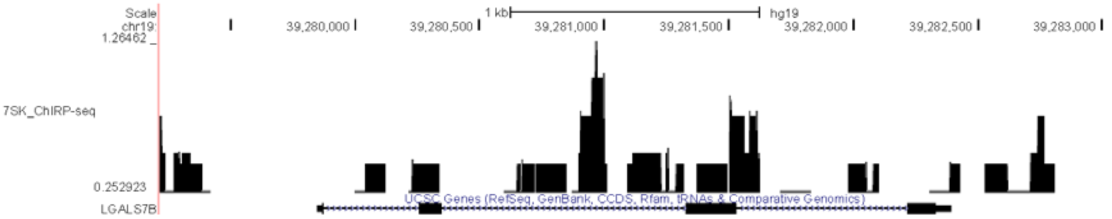

IF16: (★)

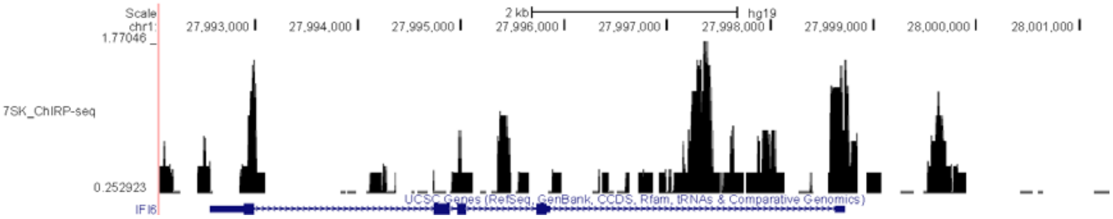

MX1: (★)

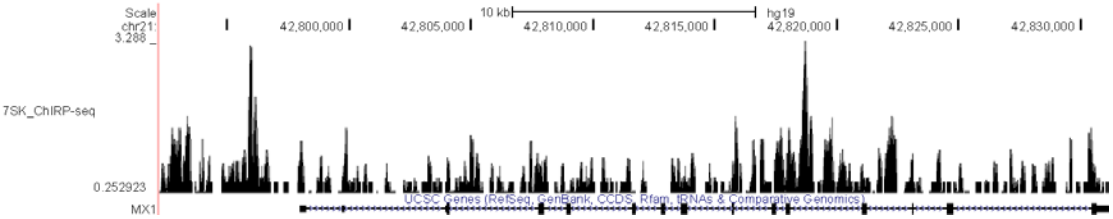

CRYAB: (★)

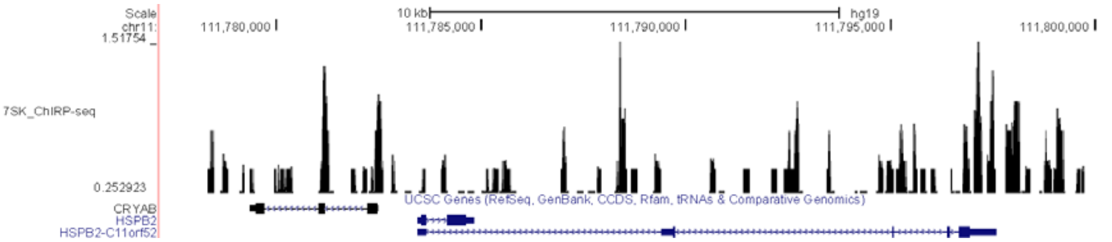

NUPR1: (★)

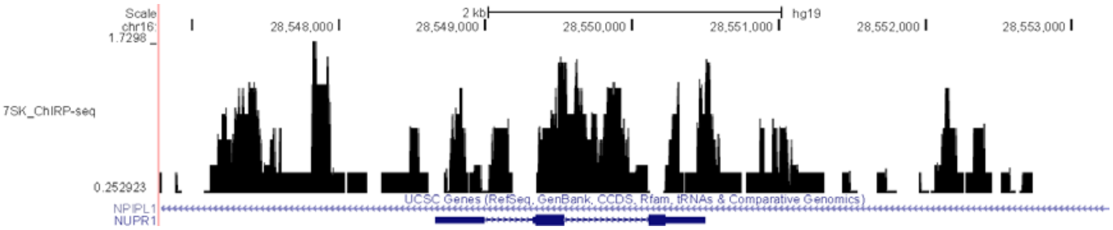

CSF3:

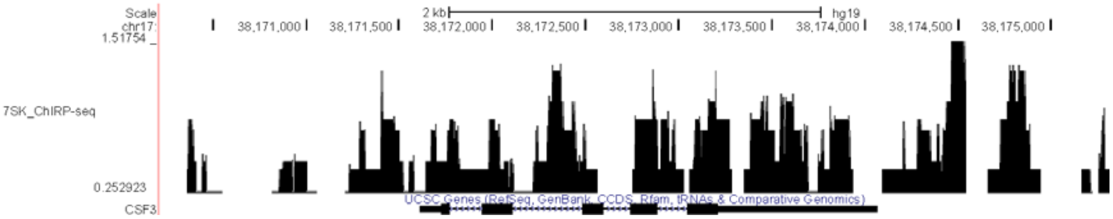

CXCL1: (★)

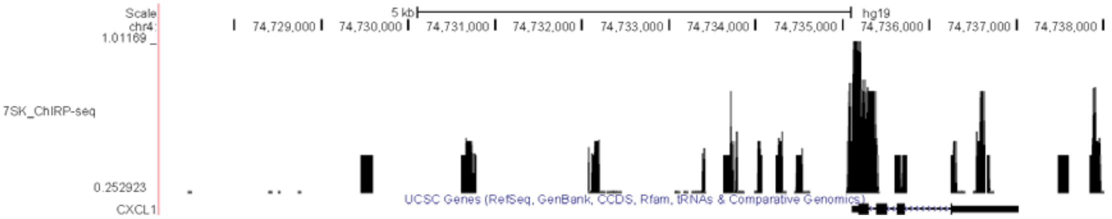

PID1:

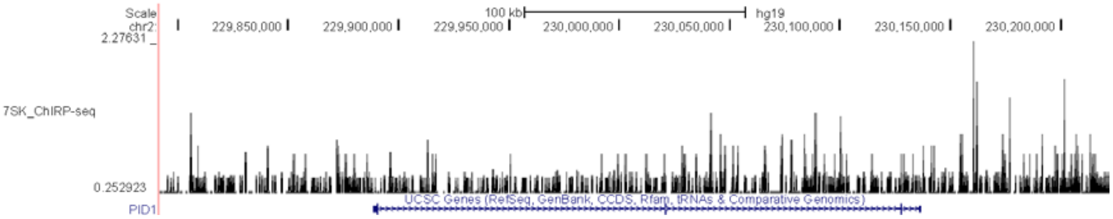

EGFL7: (★)

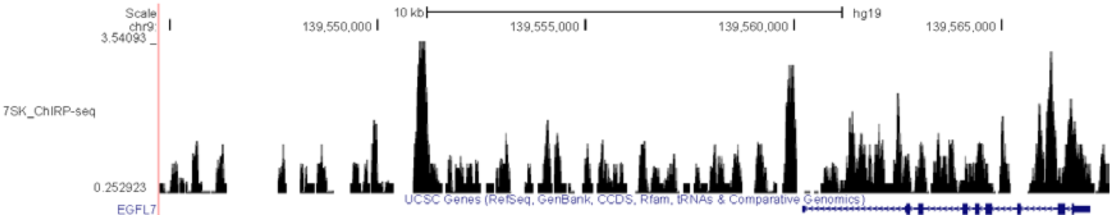

SYDE1: (★)

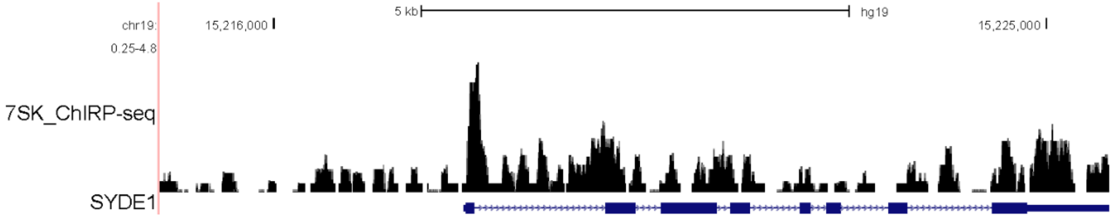

EDN2:

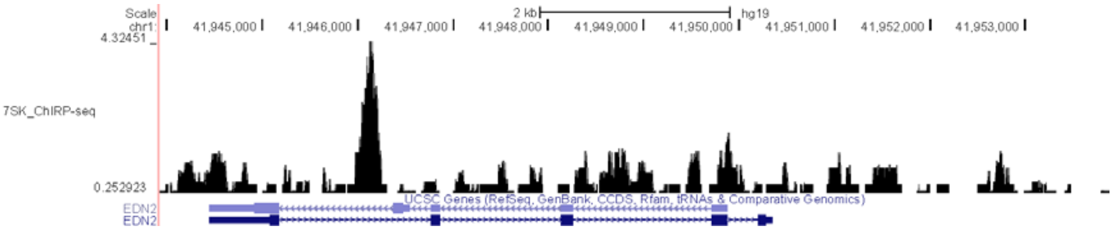

BNIP3: (★)

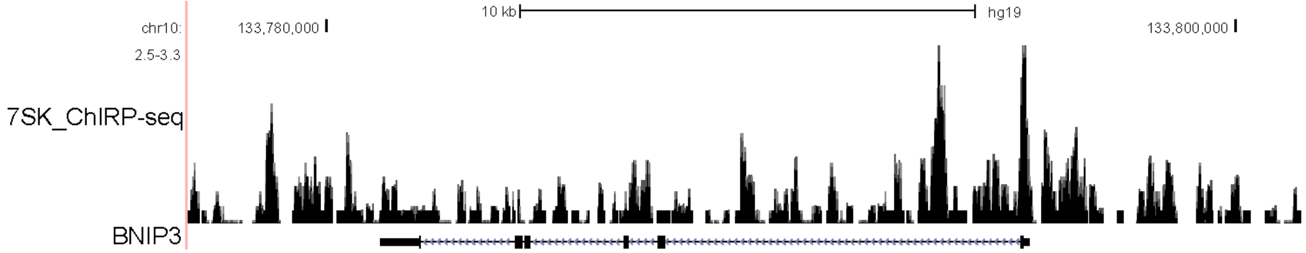

S1PR3:

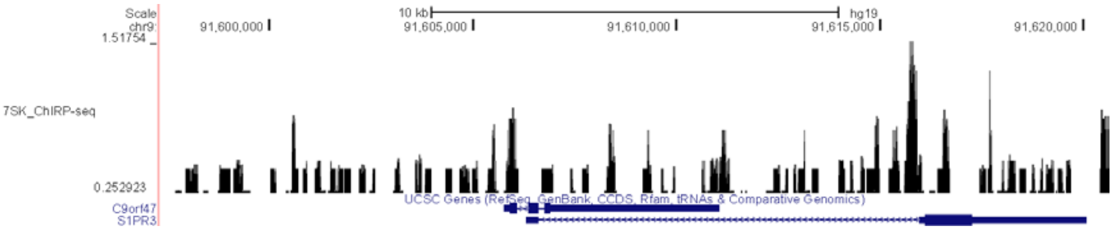

GADD45G:

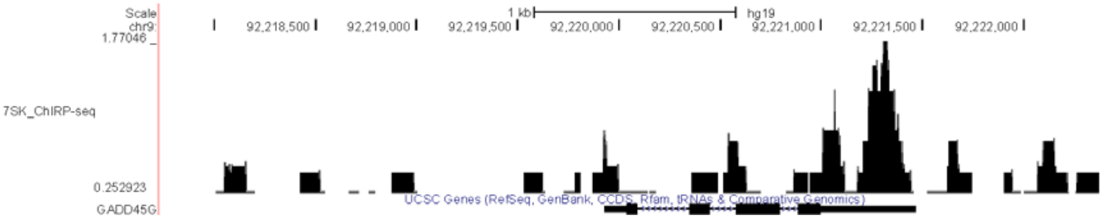

NDRG4:

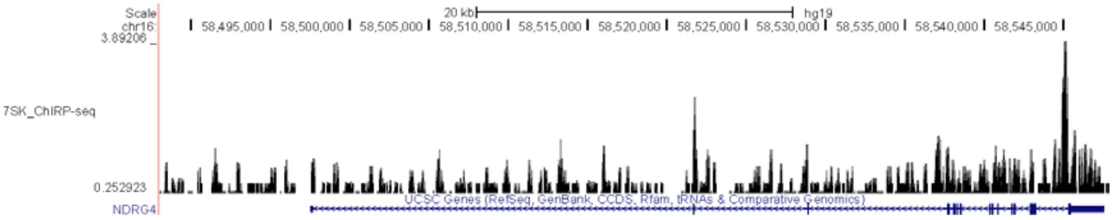

SYK:

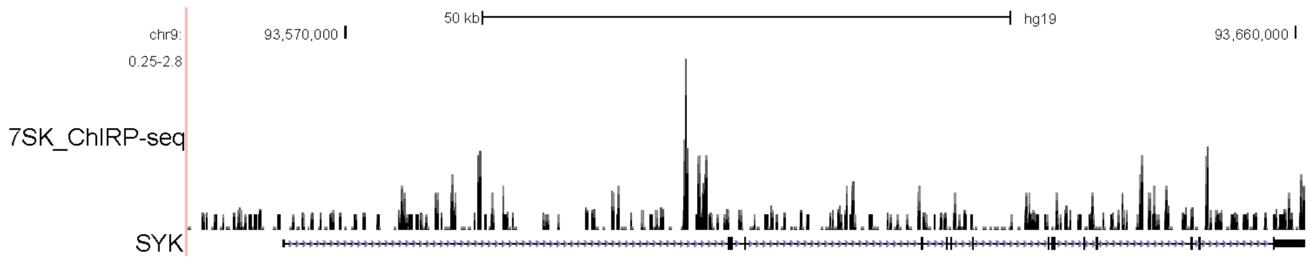

COL5A1: (★)

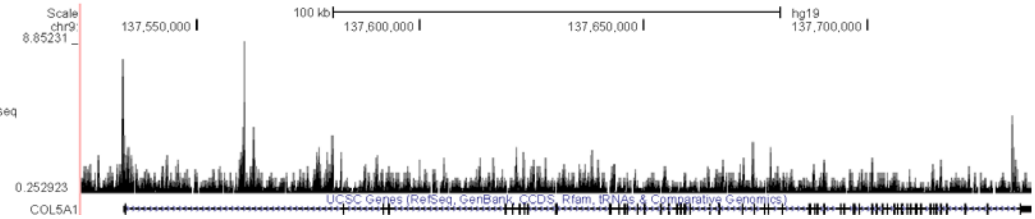

EPS8: (★)

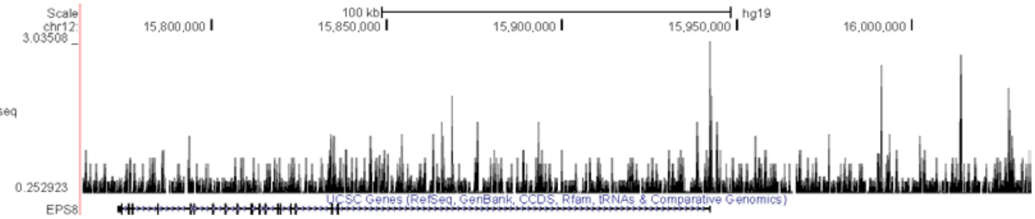

BMP4: (★)

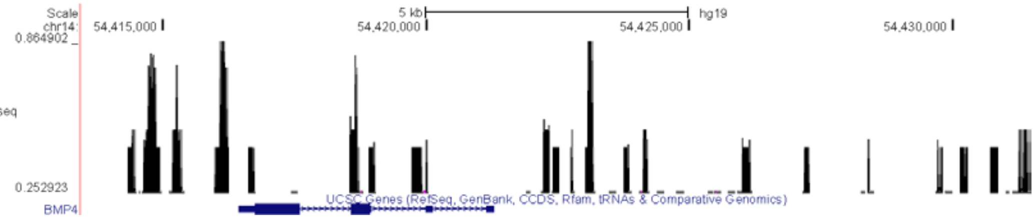

FUT8: (★)

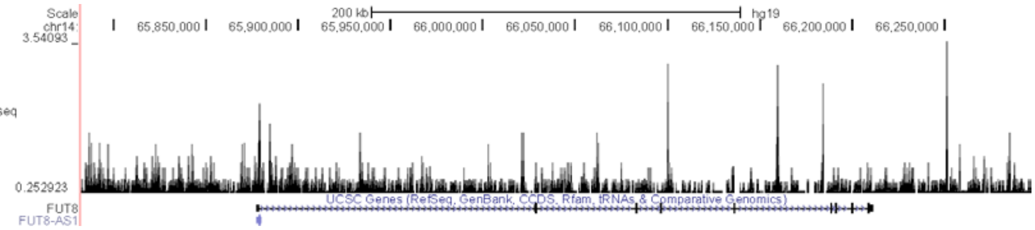

FN1:

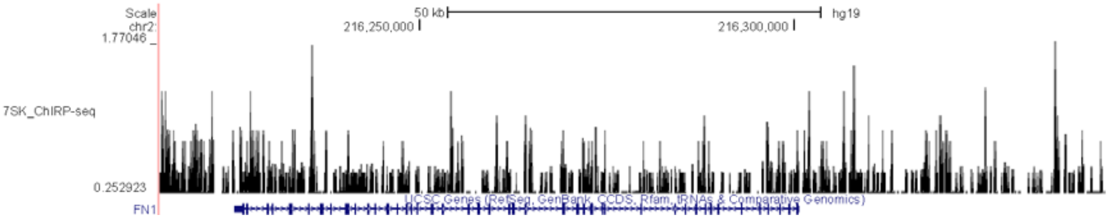

FOXG3: (★)

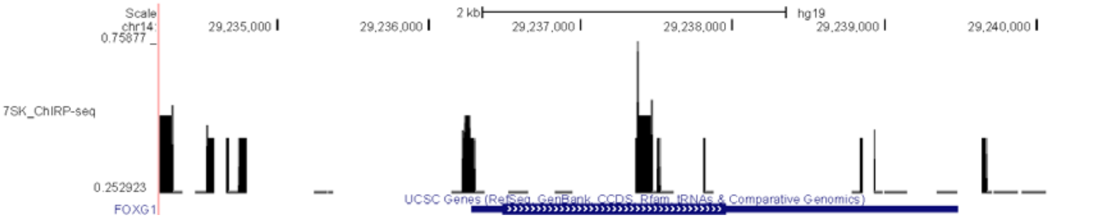

EGLN3: (★)

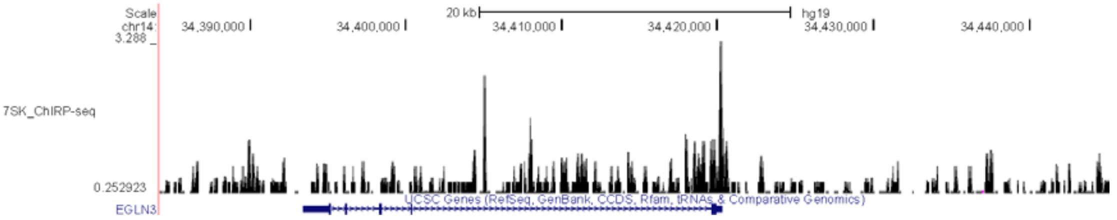

PTX3:

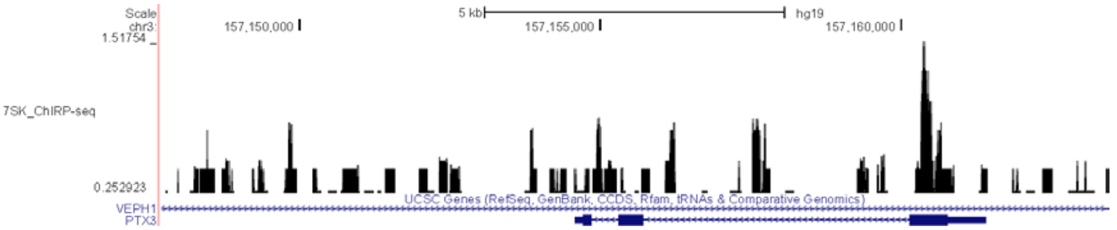

NAMPT: (★)

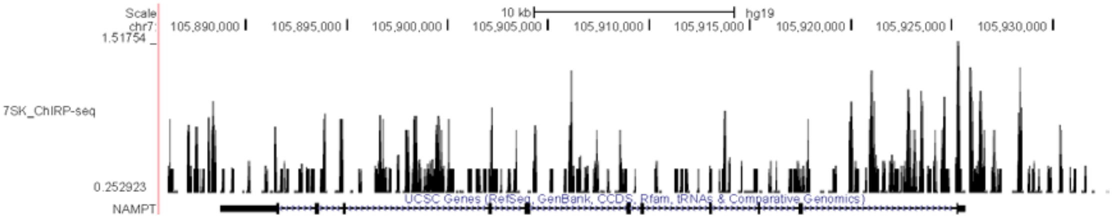

RBPMS2:

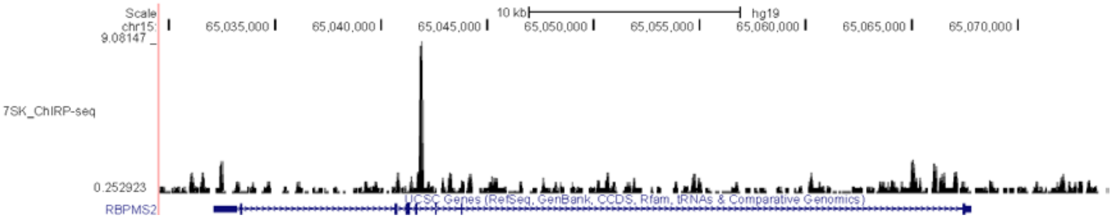

COL8A1:

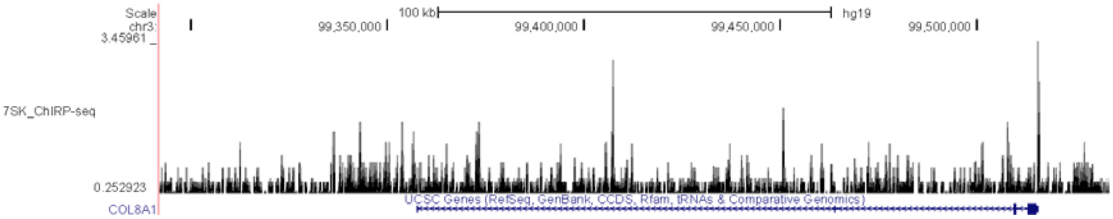

S100A8: (★)

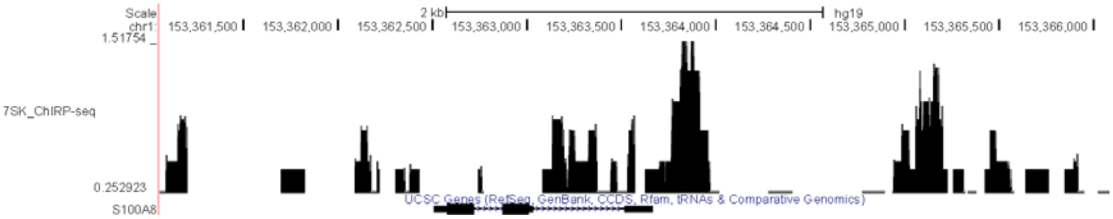

GJA1:

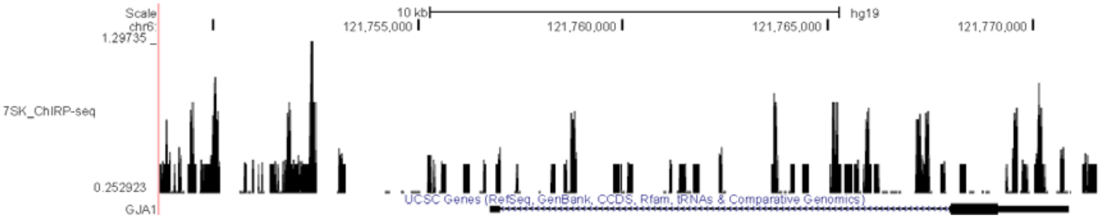

PODXL2:

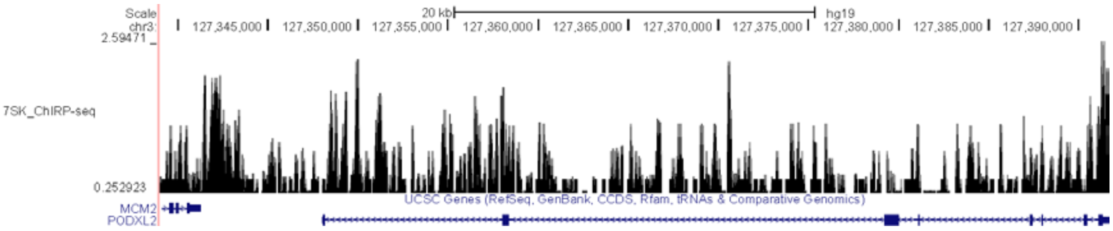

TNS1: (★)

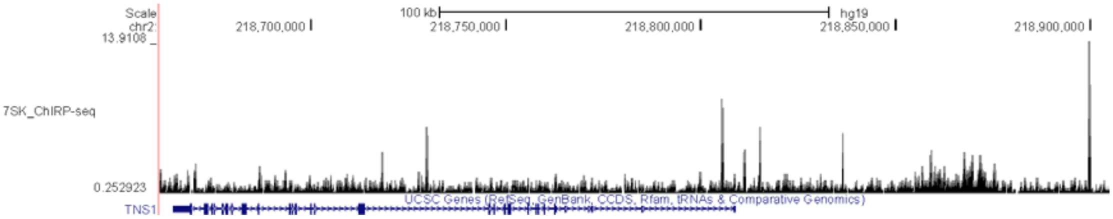

HPSE: (★)

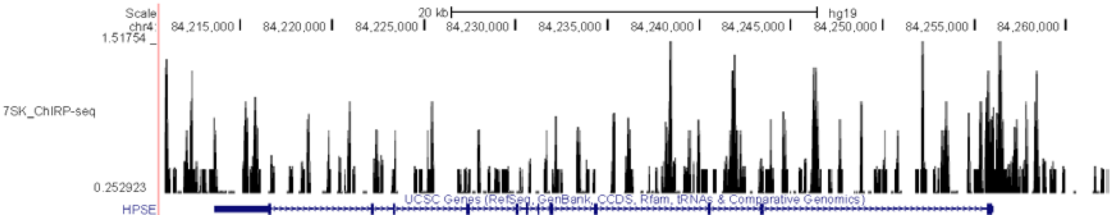

PCSK9: (★)

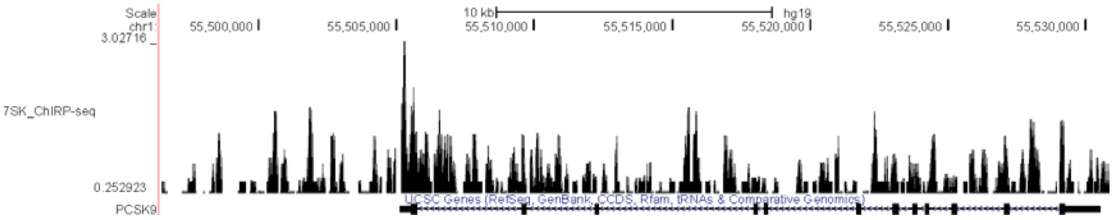

CCL2: (★)

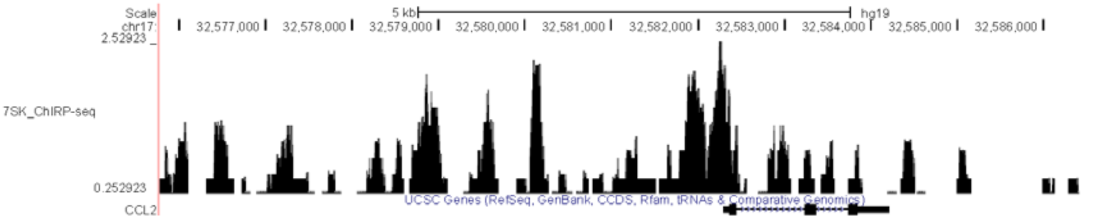

EEF1A2:

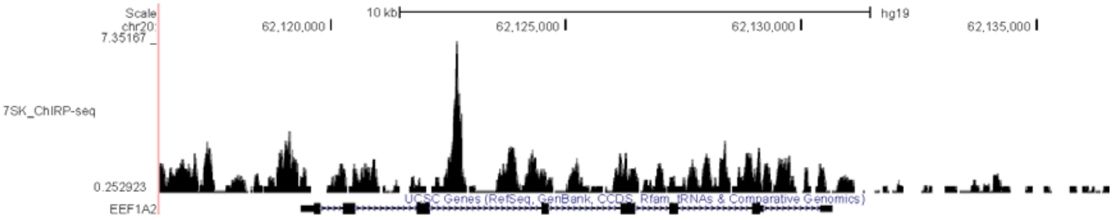

GLP2R:

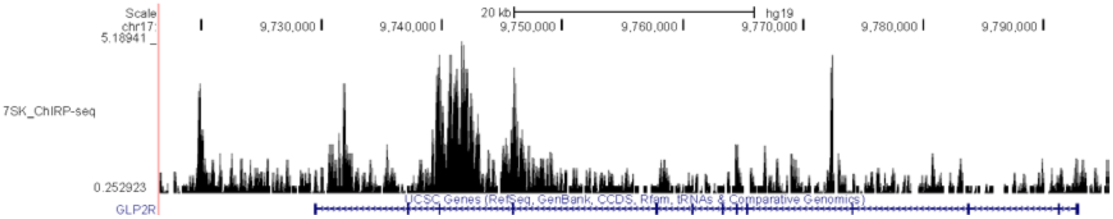

HIF1A: (★)

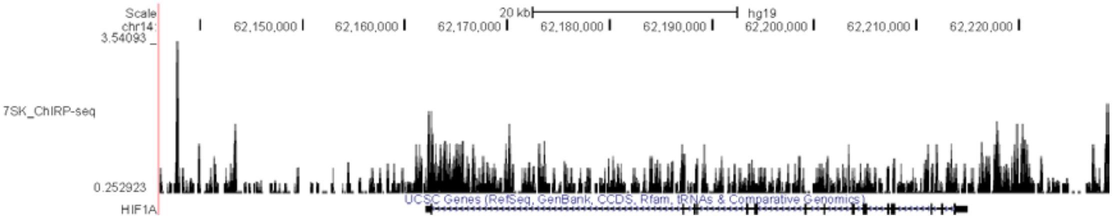

FAM162A: (★)

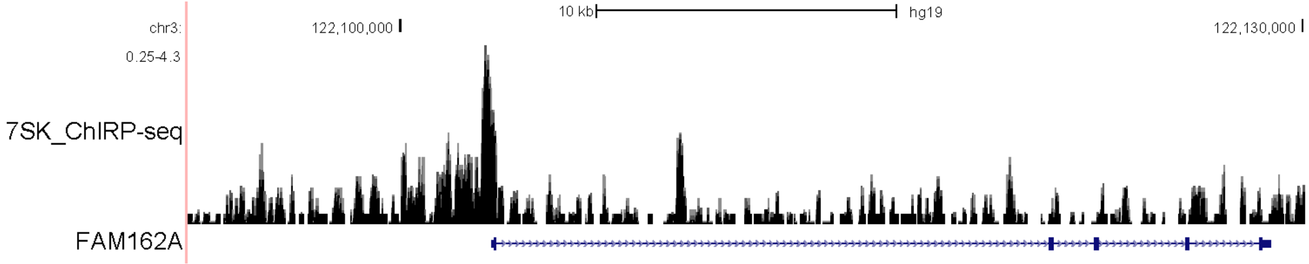

SOX18:

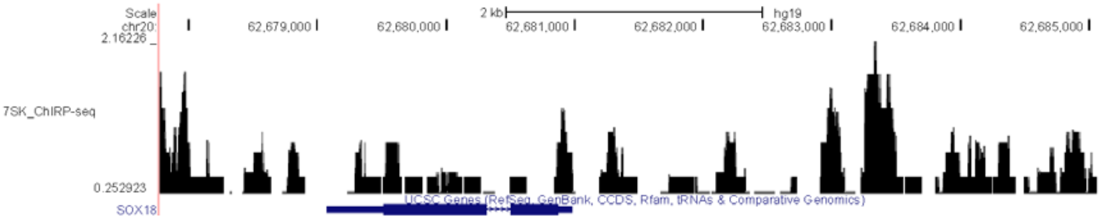

OLFM1:

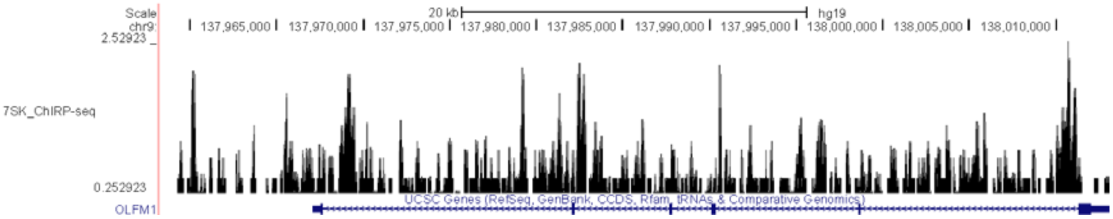

ADD2:

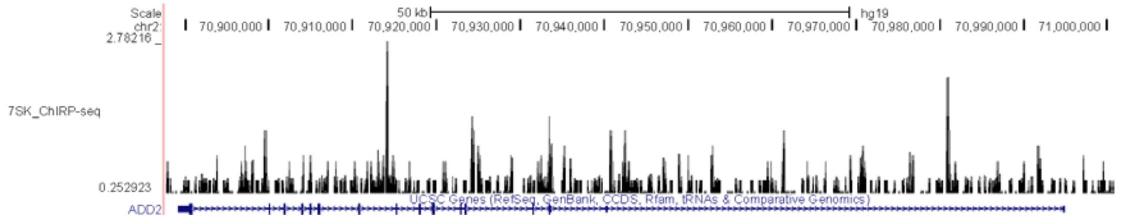

TGM2:

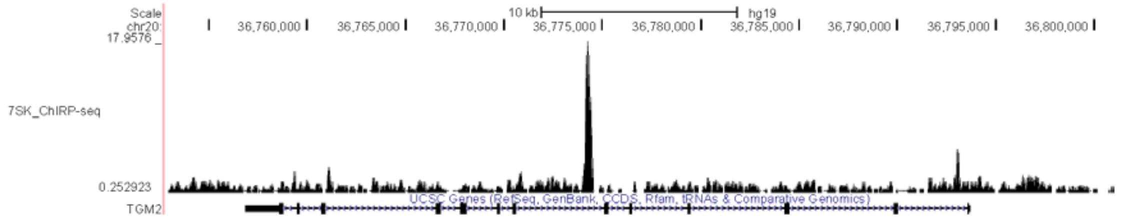

SRPX:

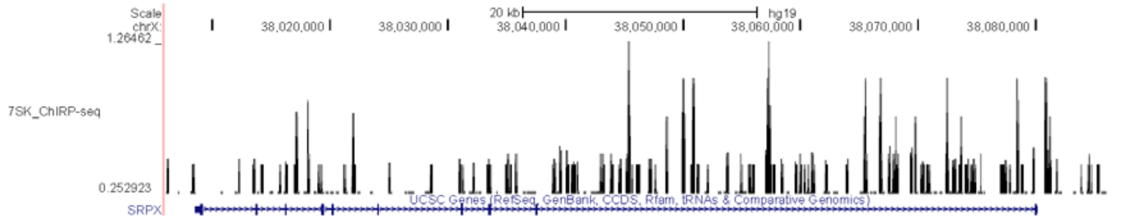

BIN1:

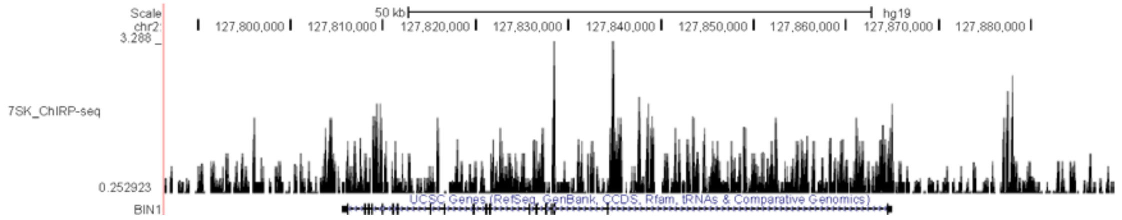

EPHA8:

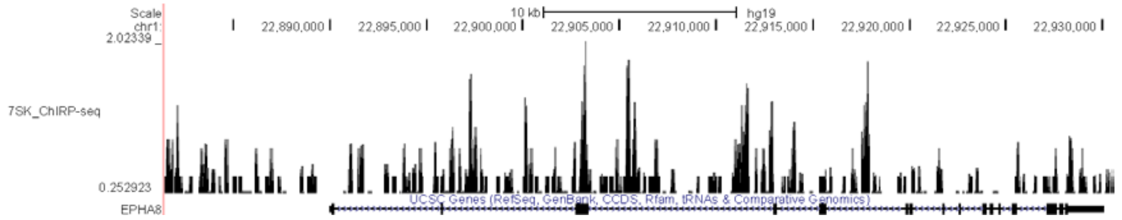

Supplementary Figure 7. 7SK ChIRP-seq data mining showed the occupancies of 7SK on 59 tumor associated genes from RNA-seq. ★ marked genes that have signals of 7SK ChIRP-seq on the promoter-proximal regulatory sequences.

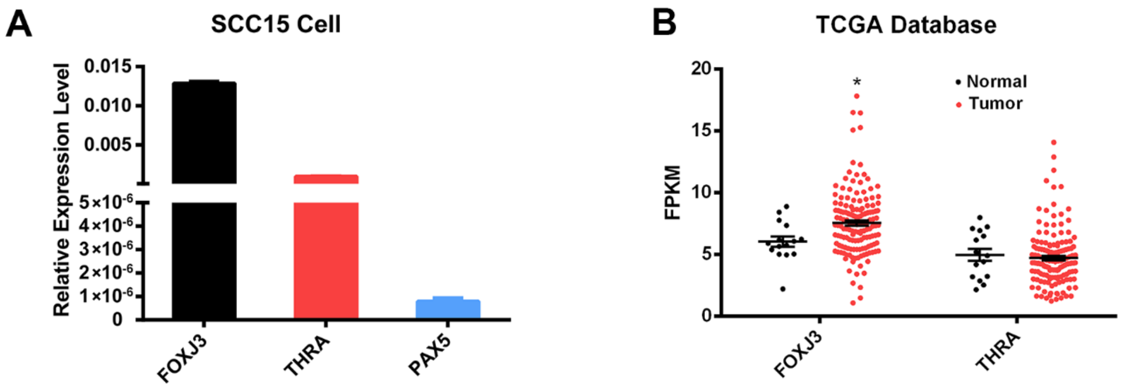

Supplementary Figure 8. (A) Expression analysis of FOXJ3, THRA and PAX5 in SCC15 cell. n = 3. **(B)** Expression analysis of FOXJ3 and THRA in TSCC patients from TCGA database (Normal = 15, Tumor = 147). *$P < 0.05$.
